# Supplementary material for: Unique Mode of Antiviral Action of a Marine Alkaloid against Ebola Virus and SARS-CoV-2
Source: Viruses. 2022 Apr 15;14(4):816. doi: 10.3390/v14040816 (PMC9028129; doi:10.3390/v14040816)
Supplement: Supplementary file 1 [file viruses-14-00816-s001.zip › viruses-1593583-supplementary.pdf]

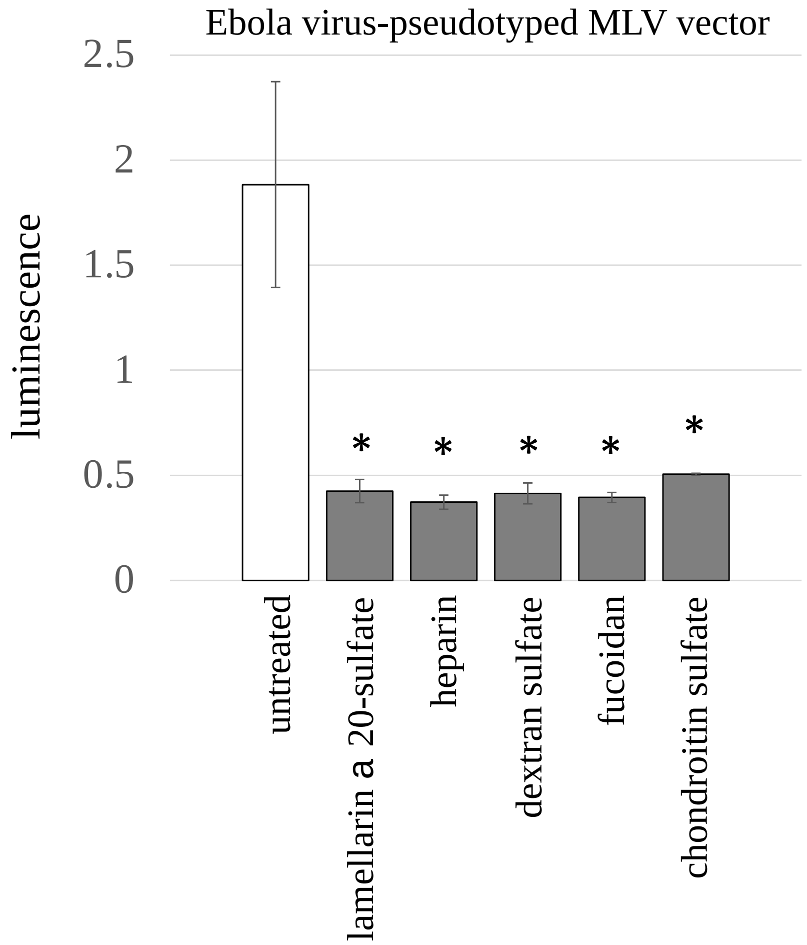

Figure S1. Impacts of lamellarin α 20-sulfate, heparin, dextran-sulfate, fucoidan, or chondroitin sulfate on Ebola virus-pseudotyped MLV vector infection. Target 293T cells were inoculated with Ebola virus-pseudotyped MLV vector in the presence of lamellarin α 20-sulfate (150 μM), heparin (50 μg/mL), dextran-sulfate (50 μg/mL), fucoidan (50 μg/mL), or chondroitin sulfate (50 μg/mL). Cell lysates were prepared from the inoculated cells. The luminescences of the cell lysates were measured. This experiment was repeated three times. Error bars indicate SD. Asterisks show significant differences compared to control cells.

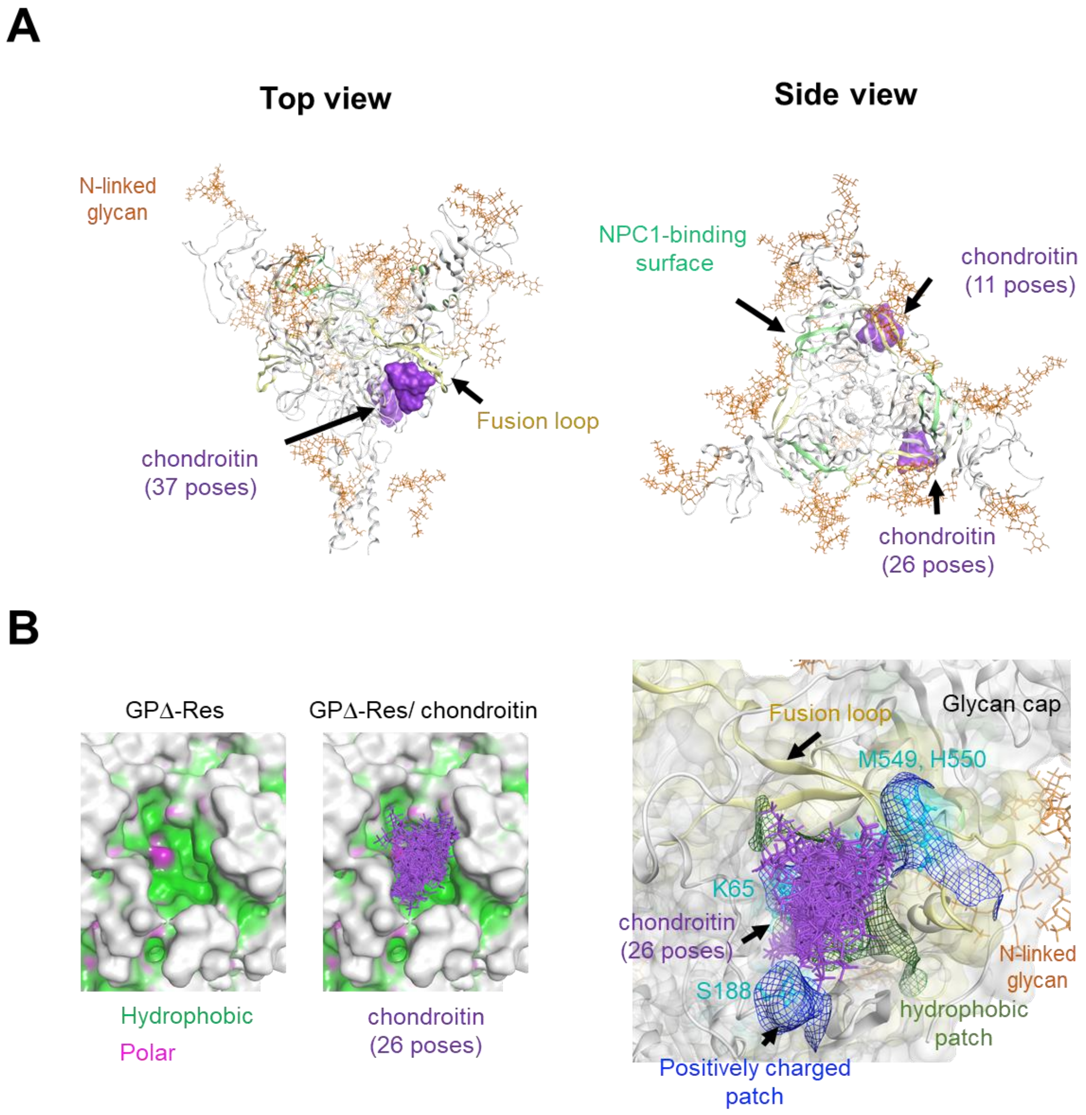

Figure S2. Superposition of the 26 docking poses of chondroitin sulfate in the principal binding site. Binding modes of the 26 docking poses of chondroitin sulfate (purple rodlike chains) in the site depicted by a green arrow in Figure 7D are shown. Chondroitin sulfate preferentially bound to a hydrophobic cavity with hydrophilic residues in portions. The binding site is suited in the fusion loop of the G2 subunit

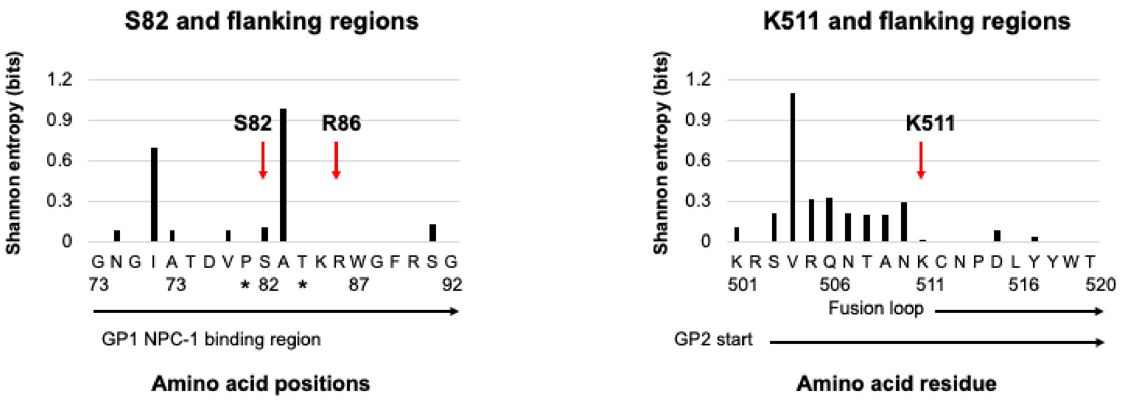

Figure S3. Variation of key residues of the Ebola virus glycoprotein for binding of lamellarin α 20-sulfate. Shannon entropy [69] was used to quantitate variations at individual amino acid positions as described previously [56,67]. Entropy scores were calculated using a total of 2,325 Ebola virus glycoprotein sequences from the Virus Pathogen Resource (ViPR) [68]. Amino acid sequences corresponding to those of the Reston Ebola virus lineage are shown. The distribution of entropy scores around S82 and K511, which play key roles in constituting the principal binding site of lamellarin α 20-sulfate (Figure 8) are shown along with amino acid positions in Reston Ebola virus (GenBank accession number: AAC54885) [43]. An entropy score of zero indicates absolute conservation, whereas a score of 4.4 bits indicates complete randomness.
